# A probe into the developmental pattern of public art education in colleges and universities from the perspective of new media

**DOI:** 10.3389/fpsyg.2023.1138950

**Published:** 2023-03-01

**Authors:** Kaile Zhang, Tsung-Chih Hsiao, Qing Tian

**Affiliations:** School of Arts, Southeast University, Nanjing, China

**Keywords:** public art education, new media, aesthetic education, IPA analysis, Internet +

## Abstract

The integration of public art education and new media in colleges and universities remains a trend which cannot be stopped. As new media has had a beneficial impact on teaching content, forms of teaching, and teaching effects, it has also created various challenges for public art education in colleges and universities. While investigating the current state of integrating new media with public art education in colleges and universities, it was found that the impact of new media benefits more than it harms, whether it be online, in-person, theoretical, or practical teaching. Based on the current state, future policy focus, the direction of new media’s changes, and the depth of cultural and art resources, new media and public art education in colleges and universities will continue to deepen integration and show further improvement. Using a survey of more than a dozen universities in the Jiangsu Province and the results of a questionnaire survey of 116 college students, this paper probes into the changes in public art education in colleges and universities in the new media era and explores the future developmental pattern of public art education in colleges and universities both internally and externally.

## Introduction

As an important means for colleges and universities to foster their students’ sense of esthetics and personality, public art education in colleges and universities has shouldered the crucial responsibility of cultivating virtues through education. How to build a public art education system in colleges and universities remains a key topic of concern for schools and people from all sectors of society. Particularly, in recent years, following China’s social attention to the influence Chinese traditional culture and art have on the thoughts and values of young students, the academic community has invested more research on the favorable effects of esthetic education in colleges and universities. During this process, new media has advanced with the times and the development of science and technology, gradually affecting every aspect of life in society. Similarly, public art classes in colleges and universities have also undergone great changes in terms of course content, teaching methods, and effectiveness.

At present, academic research on public art education in colleges and universities can be divided into several research directions: such as functional exposition, system construction, real predicaments, ways of reforming, and more. Professor Shao Ping of the Conservatory of Music, Yangzhou University, and Li Hui of the North University of China integrated the reform of public art education in colleges and universities with the development of the “Internet +” era. This paper primarily discusses the role that the Internet plays in promoting public art education and the path of teaching reform in public art classrooms in terms of its facilities, teachers, management institutions, and others (Shao, 2018; Li, 2021). Currently, however, there is an absence of research literature touching upon public art education in the context of new media changes. This paper attempts to discuss the new forms and novel features of public art education in colleges and universities in the new media era. Likewise, it attempts to consider various forms of expression of the new media, based on existing cultural and art resources and the future development and innovation trend of new media, to explore the developmental pattern of public art education in colleges and universities from the perspective of new media. This paper aims to demonstrate the positive effect that art and new media have on each other, realized through the leading role that public art education in colleges and universities has on younger students.

## Opportunities and challenges faced by public art education in colleges and universities in the new media era

“New” media has made active and two-way information interaction behavior possible, creating an information ecosystem environment where co-creation and mutual sharing are realized (Logan, 2010).

As a specialized product of thought, art cannot separate itself from the influence of social life. The booming development of the economy, the roaring progress of science and technology, and rapid transformations in the media era are bound to have a substantial impact on art as an ideological superstructure, in terms of its content and form, its creation and appreciation. Public art education in colleges and universities also presents different forms and features of expression under the developmental pattern of new media, resulting in positive or negative effects, as it is faced with unprecedented opportunities and challenges.

### Leapfrog development of teaching content, forms of teaching, and effectiveness of teaching

#### The abundance of teaching content

In the new media era, public art education in colleges and universities has undergone changes in terms of the content of its curriculum. This change should first be attributed to the inevitable encounter of new media and art in these times. As science and technology profoundly impact society, art has collided with technology. Changes in the social environment of artistic creation, the creative methods and means of art, and the emergence of new categories of art are all advancing at the speed of sound. Public art education in colleges and universities has also created new content for curriculums, supplementing “traditional” cultures and art styles of the East and West. Examples of this are photography, with the camera as its creative medium; film, which came about at the end of the 19th century as the eighth art; and all kinds of new media art forms arising during the transformation of media: video art, virtual art, interactive art, digital art, and more (Cho, 2013). These newfound art categories and old art created by novel methods not only add to the meaning of “art” but also allow a new generation of young students to see more possibilities of “art.” Students have more of a variety in art courses or in extracurricular art practices and career planning.

#### Diversified teaching forms

In addition to the classroom content, the greatest impact new media has on public art education in colleges and universities stems from how the curriculum is taught. Firstly, by incorporating the distinct characteristics of new media breaking the boundaries of time and space, and realizing two-way interaction, public art courses in the new media era can be conducted in an online classroom. Here, students can also acquire knowledge without being limited to only in-person lectures. Secondly, teachers can employ a variety of multimedia software and hardware and integrate this with verbal teaching, hands-on guidance, and other teaching methods that present a myriad of content. Likewise, students can readily acquire more comprehensible art resources among the vast information on the Internet, which will equip them with a more concrete understanding of a course’s content. When the richness and cutting-edge aspects of new media become integrated into public art classrooms in colleges and universities, the modality of public art education can have room to expand, enabling the convenience of technologies to fully display the charm of art (Barakina et al., 2021; Wu et al., 2022).

#### Prominent teaching effects

Involving new media forms in public art education in colleges and universities has produced a never-before-seen effect in the classroom. In a survey of 116 college students, it was found that 72.4% of the respondents believed that abstract curriculum content and knowledge could be made concrete by applying forms of new media in public art classes (as shown in Table 1), ultimately stimulating the students’ interest in learning. This dovetails the specific requirements of art appreciation: art needs to be personally experienced and appreciated, and the beauty of works of art and the peak experience gained from art can seldom be obtained by telling from others. When the Internet, electronic devices, and XR technology bring the “Creation of Adam,” a mural by the brilliant Renaissance artist, Michelangelo, from thousands of miles away to the classroom, students can take a “trip” to the Sistine Chapel. This can do much to pique their interest in learning and deepen their understanding of art. More notably, the current development trend of new media is moving in the direction of augmenting physical perceptions, thereby realizing an “immersive” experience. For instance, along with the birth of new media forms such as 3D, 4D, VR, AR, and others, the forms that public art education will have at its disposal will be increasingly abundant, enabling teaching effects to progress by giant strides (Hudson et al., 2019).

**Table 1:** The favorable influence of new media in public art classrooms.

Elements	Frequency	Proportion
Materialize the theoretical knowledge	84	72.4%
Stimulate interest in learning	69	59.5%
Constraints of discipline	56	48.3%
Expand access to art resources	47	40.5%
Enrich the forms of art practice	38	32.8%

### Troubles arise from “pan-entertainment” and “artistic damage”

While public art education in colleges and universities has produced positive changes in the new media era mentioned above, adverse effects have accompanied new media, bringing challenges to art education that colleges and universities need to confront (Zhang and Yang, 2022).

**Figure 1:**
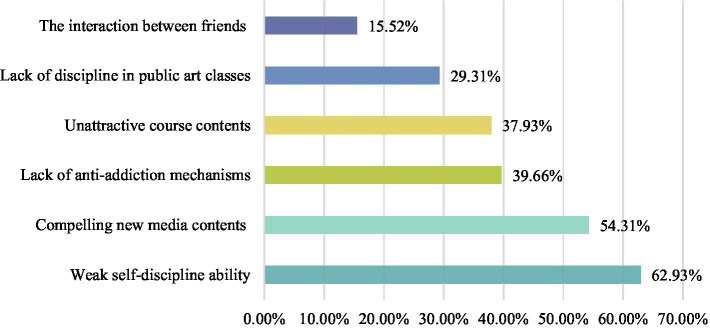
The cause of inattention in public art classes.

Firstly, the profusion of products and content coming from the Internet can be overwhelming. Public art classes need to be able to identify the validity of information on the Internet while benefiting from its convenience. Confronted with a “pan-entertainment” Internet environment, college students need to be able to resist enticement, differentiate high-quality information, and actively participate in public art classes, although this requires a high level of self-discipline from the students (Cao, 2019). In the survey, some 60% of the students said that they had issues paying attention in public art classes because of the influence of new media. More than 1/3 of the students attribute this lack of attention to unappealing course content; meanwhile, more than half of the respondents think that online content is riddled with temptations. This, coupled with the lack of self-discipline, leads to inattention in public art classes (see Figure 1). It is worth noting that, different from the high-intensity assessment requirements of professional courses, students believe that the content of public art courses is irrelevant to graduation and ignore the self-control ability of “delayed gratification.” Therefore, students’ weak sense of self-discipline is more obvious in the public art classes. The ubiquity of this phenomenon necessitates public art education to devote more energy to curriculum design, content planning, and selection of teaching modalities. This can provide students with useful art knowledge in a form that students enjoy taking in, limit students’ behavior in a tough or flexible manner, and draw the attention of the students to the curriculum content, which will maximize the object of public art education of cultivating students’ sense of esthetics and improve their esthetic ability and proficiency.

Secondly, in a way, new media opens up a wide communication path to art, enabling the comprehensive popularization of art. However, new media that is replete with false information and apathetic reason runs counter to an art that is infused with deep thought and tender sentiments, and that requires the individual to fully invest themselves when experiencing it. In this context, it becomes difficult for traditional art, particularly represented by Chinese paintings, to reproduce that same charm obtained by the viewer seeing it with their own eyes in person, damaging the uniqueness, autonomy, and authenticity of art in the new media era (Innocenti, 2015). Accordingly, confined by the development of new media at this stage, it remains difficult for public art education in colleges and universities to break through the shackles of technology, as it remains difficult to reproduce the same effect that traditional art has when it is taught through methods orally, hands-on, and tacitly understood.

## The realistic development of the integration of public art education and new media in colleges and universities

Currently, public art curriculums in colleges and universities are devoted to expanding the breadth and depth of their influence, virtually doing away with the previously dull and uninteresting curriculum and teaching arrangements of before, as they try to combine local resources and strive to showcase more innovative content. New media plays an essential role in this process. When studying online or in-person, theoretical or practical courses, new media can be deeply integrated into art classes in terms of benefiting teachers and students, arousing students’ enthusiasm, and furnishing vast amounts of information. This helps public art education in colleges and universities cultivate students’ ability to discover, appreciate, and create beauty.

### Two-pronged approach for in-person physical and online network

By surveying more than a dozen colleges and universities in the Jiangsu Province, we know that at present, most colleges and universities have not limited their focus on public art education to in-person physical teaching. Considering the current shortage of teachers in public art education and the uneven distribution of qualified teachers, colleges, and universities have joined online teaching platforms to share high-quality and public online art courses. There are many online teaching platforms, such as the Chinese University MOOC (Massive Open Online Courses), Chaoxing Erya MOOC, NetEase Online Open Courses, XuetangX, and more. Their course resources are comprehensive, rich, and high in quality (Lin et al., 2015). Art masters from top professional colleges and universities impart art knowledge through these online classes and benefit countless young students from around the globe, around the country, and around colleges. Several courses involve general art theories and art principles, to art appreciation and art criticism, spanning a variety of fields based on the defining characteristics of various art disciplines. These courses have included: “Listening to Music” by Professor Craig Wright of Yale University; “Meaning in the Image: Appreciation of Chinese Classical Poetry” by Professor Yu Dan of Beijing Normal University; “Art History” by Professor Zhu Qingsheng of Peking University; “Talking about Film” by Professor Dai Jinhua of Peking University; “Art Philosophy and Aesthetic Problems” by Professor Wang Defeng of Fudan University; “Principles of Art and Art Appreciation” by Professor Yang Qi of Tsinghua University; and “An Introduction to Art” by Professor Peng Jixiang of Peking University. These courses have afforded a variety of knowledge and content for students with various needs. By combining it with the in-person curriculum arrangements of colleges and universities, it hopes to popularize art knowledge, expand students’ learning channels, and promote the dissemination of art education resources (Enyu, 2018; Ma, 2021).

New media expands the channels for students to enjoy art education resources through online courses. For in-person physical classes, the impact of new media on public art education is mainly reflected in teaching equipment and teaching methods. As aforementioned, art as a special form requires the individual to fully invest themselves when experiencing its esthetic connotation. Public art education in colleges and universities needs to do the utmost when creating an “atmosphere” for students to appreciate art and this is the role of new media (Örtegren, 2012). When the students in the class hear Beethoven’s “Destiny” and Debussy’s “Moonlight,” the differences between these composers’ thoughts, personalities, and circumstances can be conveyed more clearly. When students see “The Death of the Marat” by neoclassical painter Jacques Louis David and “The Swing” by the Rococo artist, Fragonard, on their screen, they will be able to better understand the differences in artistic features of western art in different time periods and in different contexts. As the Chinese folk dance drama “Along the Silk Road” and the ballet “Swan Lake” are vividly displayed in class, it becomes more intuitive and comprehensible for students to comprehend the differences in dance art under the influence of Eastern and Western cultures. On the one hand, if the explanation of art-related knowledge depends solely on oral teaching, in some ways, it cannot really convey the meaning and value of works of art. On the other hand, this also makes it difficult to pique the enthusiasm of students when learning theoretical knowledge and hinders the effectiveness of teaching. So, the integration of new media can fill this gap.

### Theoretical learning and practical exploration go hand in hand

Currently, according to policy requirements, while most colleges and universities strive to be comprehensive and thorough when setting up their curriculums and combine online and in-person teaching methods, they still strive to integrate theoretical study and practical exploration into the courses. When students go out of the classroom, they can apply theoretical knowledge to practical exploration, letting theoretical knowledge guide their practice and deepening theoretical application in practice. Likewise, when incorporated with their own majors, this method of learning can expand their thinking and give full play to their spirit of innovation, allowing the power of art to flow through their professional studies. The study of artistic knowledge can even affect the way students consider problems, as a spirit of estheticism can change an individual’s attitude toward life. The role that new media can play in this process is primarily shown by providing a broad reserve of resources and enriching the categories and forms of artistic practice.

First, there is a huge demand for reference literature when studying art theory. When physical resources in public art education libraries of colleges and universities are not readily accessible, online literature resources become even more valuable. New media has greatly improved both electronic literature resource displays and cloud literature databases. We can readily access literature on laptops or even our mobile phones, as there are many platforms, such as Chaoxing learning APP, CNKI, Z-Library, and others. Furthermore, specialized art resources are becoming more and more complete, and museums and exhibition halls have set up their own online resource libraries influenced by the concept of interconnection in the new media era. Examples of this are the official website of the Palace Museum, the Chinese Treasure Museum, and the British Museum. Students can promptly find the content they need through these corresponding channels. This plays a positive role in students’ independent understanding, learning, and innovation, as well as in cultivating students’ ability to think and explore on their own (Rohotchenko et al., 2021).

**Figure 2:**
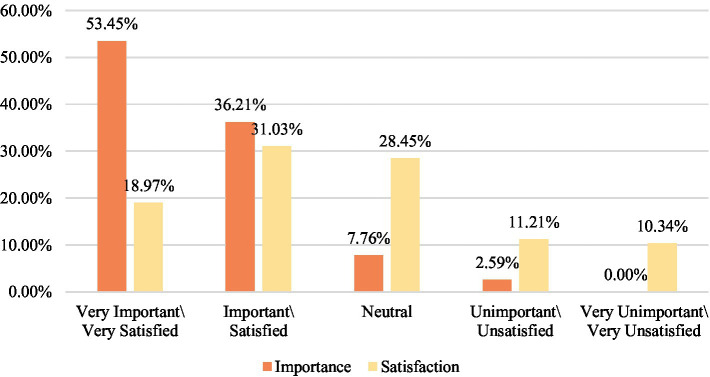
Significance and satisfaction level of the interactive feature of new media in public art classes.

**Figure 3:**
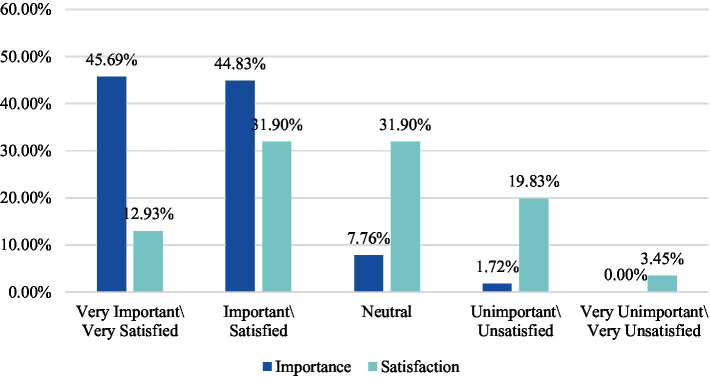
Significance and satisfaction level of new media art creation in public art classes.

Second, the practical curriculum of public art education in colleges and universities in the new media era has a wider range of art categories with less complicated operations. Students can use the equipment at hand to complete their artistic creations in photography class, film class, and new media art class. Mobile phones, digital SLR cameras, notebooks, tablets, VR headsets, and other devices can be used as instruments for students to showcase their artistic ideas and become a stepping stone for students to embark on the road of art. Likewise, the applications of new media are becoming more prominent during field trips to museums, galleries, and other in-person activities (Sujin, 2018). Many museums around the world have recently incorporated new media elements into the design of their exhibition halls. Curators will also employ various hi-tech methods to make the exhibition content more fascinating while enhancing the audience’s sense of participation and achievement. Learning in more fun and entertaining ways plays a considerable role in boosting young students’ willingness to understand the traditional culture and art (Tim et al., 2020). Nonetheless, we have also found that such a diversity of art practice and art creation methods is oriented toward a minority of people, with a number of colleges and universities being more concerned with outputting theoretical knowledge when integrating new media into public art classrooms. This absence of applied new media for art creation and art practice courses takes away from the content aimed at boosting students’ participation. According to the results of the survey, students value being able to interact with new media used in public art classes and having the opportunity to use new media in their artistic creations, but are relatively dissatisfied with the current situation (as shown in Figures 2, 3). Art practice is not only an integral part of public art education, but also an important means to have students pay more attention to the curriculum content. This is why the integration of new media and public art curricula must pay greater attention to the creation of practice and interactive content.

## The future developmental pattern of public art education in colleges and universities from the perspective of new media

In modern society, new media has affected virtually every aspect of daily life, making the integration of public art education and new media an unstoppable trend. Although new media furnishes new energy to public art education, we must not ignore the downsides of new media. Nonetheless, the attention drawn from relevant government departments, the booming development of new media, and the profound heritage of traditional art are all aspects that make us look forward to the future development of public art education in colleges and universities. The future development of public art education in colleges and universities should also be designed around the following dimensions based on various conditions: fully utilizing opportunities for development in this era and society; endeavoring to realize the popularization of art education originating from colleges and universities; and letting the power of art permeate the development of society.

**Figure 4:**
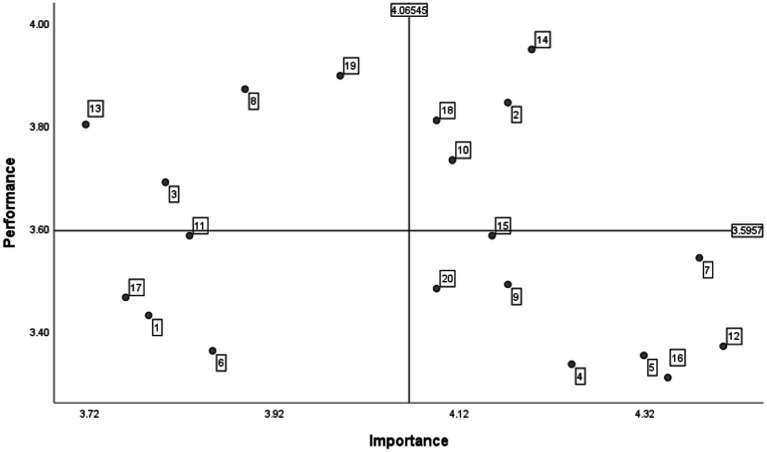
IPA analysis of satisfaction on public art classes in colleges and universities in the new media era. (1) The accessibility of public art education policy in new media; (2) the support of policy on public art education for the integration of new media and public art classroom; (3) the degree of implementation of public art education policy; (4) the degree of freedom of the upward feedback of practical issues; (5) the richness of new media resources; (6) the degree of new media’s allocation of cutting-edge resources; (7) the degree of interest and appeal of new media used in public art classrooms. (8) the degree of adaptation of the new media used in the public art classroom to the curriculum content; (9) the variety of the forms of integration of the public art classroom and the new media; (10) the degree of convenience when obtaining public art courses in other colleges and universities; (11) the familiarity of public art teachers with the new media used in the classroom; (12) the degree of interactivity of the new media used in the public art classroom; (13) the level of limitations by public art teachers on students’ use of new media in the classroom; (14) the use of new media in public art classes helps individuals to increase their knowledge of art; (15) the use of new media in public art classes helps individuals to gain a sense of pleasure and have a peak art experience; (16) students use new media to create art in public art classrooms; (17) the degree of innovation in the evaluation and assessment of public art courses integrated with new media; (18) the depth of national traditional culture and art in the content of public art courses; (19) the degree of combination of public art curriculum content and social core values; (20) the degree of national, regional, and distinct elements used in public art curriculum content.

Considering these dimensions, this study has conducted a survey on 116 college students’ satisfaction with public art classes in the new media era. By separating these three dimensions into 20 elements, the respondents get to rate the importance and satisfaction of each element, respectively, culminating in the results shown in Figure 4. Overall, the research object of this survey is college students, who directly experience public art classes and the integrative effects of new media and public art curriculum. Likewise, their needs should be prioritized when designing the future developmental pattern of public art education in colleges and universities from the perspective of new media. Accordingly, by incorporating an IPA analysis of college public art classroom satisfaction in the new media era, this study makes the following analysis of the future developmental pattern of the integration of new media and public art education in hopes of laying a theoretical foundation for making substantial achievements in the public art education sector.

### Allowing policies to lead and actions to “guide”

#### Premise: Incorporating into “globalization” and changing the game with “soft power”

In modern society, under the influence of the Internet and other new media, living in a “global village” has suddenly narrowed the distance between various countries. While this developmental trend has come with vast opportunities and countries’ political, economic, and cultural barriers have been somewhat broken through, the difficulties we face remain increasingly complex. However, if we wish to realize development, it is best for the country not to adopt a closed-door policy. Similarly, how to avoid such risks has become a crucial factor for countries when competing. A country’s comprehensive national strength is no longer measured by its military prowess or economic strength. The “soft power” in ideological sectors including culture and arts carries a more profound and long-lasting effect. Largely, it has become a key factor for a country to break through the confines of “globalization” (Altugan, 2015). As the future leaders of the new era, it is particularly important for young students to have a positive ideology and equip themselves with the necessary knowledge. Thus, the role of esthetic education has become increasingly prominent. And art, especially traditional art, permeating the blood of each nationality, is an integral link in the development of esthetic education in colleges and universities. It carries great significance when cultivating the ideology of the youth. In other words, new media provides a shortcut for the implementation and development of public art education at the national level. By making good use of the convenient, interactive, and two-way communicative aspects of new media, information interaction and cultural exchanges between and within countries become more efficient, as the scope for choice enlarges.

#### Fundamental: Stress the functions that government plays in “guidance,” “intervening,” and “giving free rein”

**Figure 5:**
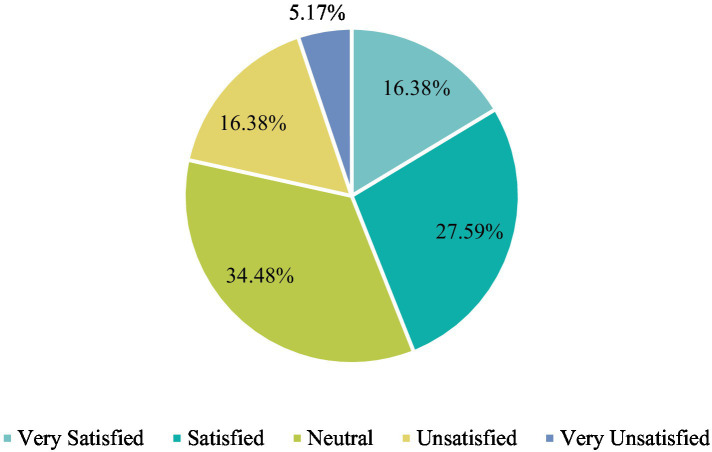
Satisfaction with the degree of unimpeded feedback on practical issues in the class.

In the survey, it was found that currently, the public remains dissatisfied with the ease of obtaining public art education policies in colleges and universities in the new media, with more than half of the respondents being satisfied with this; meanwhile, while students attach great importance to the free flow of feedback to higher authorities when encountering practical problems during the curriculum, they remain relatively dissatisfied with how things are (as shown in Figure 5). Without a feedback mechanism, decision-makers, executors, and influential individuals cannot realize basic communication, leading to a deviation of timeliness and effectiveness when integrating new media and public art classrooms. This ultimately affects the role that policies play.

What kind of role should government departments perform in the development of public art education? How to make use of the various functions of government organs to promote an improved and more rapid development of public art education in colleges and universities? How to improve the government’s capacity for public management in the new media era? How to utilize new media’s fast feedback feature to engage more active government management? Firstly, the superior functional departments need to clarify the importance of art education and its popularization by giving priority to it, particularly the art education of young students. Secondly, to instruct the direction, goal, and boundaries of the implementation of public art education in colleges and universities *via* binding and guiding provisions such as agendas, policies, plans, laws, and regulations, and to set up a feedback platform. Using new media’s two-way communicative property to receive feedback and investigate its state of implementation, and promote or amend the provisions accordingly. Lastly, afford local governments and universities enough room for independent adjustments and innovation. Under the guidance of a proper direction and framework, local universities should be able to make some adjustments according to their specific conditions, creating a public art education program with distinct characteristics (Shaw, 2020). In summary, competent government departments should neither neglect the development of public art education in colleges and universities, nor excessively interfere with and restrict its diversification. Based on the principle of “guiding,” they should incorporate functions of “restraint” and “supervision,” and help to find a development path for public art education that seeks common ground while reserving differences between local governments and universities.

#### Guideline: From central to local, from culture to art

After developing a concept of employing soft power to break through the confines of economic globalization while elucidating the role that government departments play in “guiding” the development of public art education in colleges and universities, the lines for how government’s policies should coordinate with other policies, and how these policies should coordinate with concrete measures ought to permeate down from central to local governments, as its content should permeate down from culture into art. First, in terms of the formulation and implementation of policy lines, after becoming familiar with the overall situation of the development of public art education in colleges and universities, relevant central departments should formulate applicable policies under the principle of “control and guidance.” Likewise, this should fully demonstrate the central government’s functions of organization, coordination, control, and supervision, paving the way and shining a light on the development of public art education in colleges and universities in an economic, political, social, and cultural sense. This will not only demarcate the boundaries of development, but also provide local governments and universities with a wider range of freedom within these boundaries. Grounded on a centralized spirit, local governments at various levels and the main entity of public art education (colleges and universities) should aim to be more detailed when implementing their work and applying other innovative methods, while also considering the local conditions. This will put the cause of public art education in colleges and universities into effect, bringing prosperity in a multidimensional and comprehensive manner. Second, in terms of the substance of the policy, central and local governments at each level should spearhead cultural endeavors and cultural industries, utilizing the visual aspects and entertaining nature of art education, which will harness public art education as a prominent force in the development of culture and education. This allows the creativity and sentimental charm of art to amplify the influence of culture on young students.

### The iteration of media and the courage to innovate

#### The key is to stay abreast with the rise of the media and create a dynamic classroom

In the results of the survey, students were not satisfied with the variety and cutting-edge aspects of new media resources in public art classrooms. This indicates that government departments and universities do not have a deep enough understanding of the relationship between new media and the public art classroom. In addition, capital investments and the quality of hardware equipment still fall behind. In terms of its integration, the appeal that students have toward this integration, their view of the integration’s variety and richness of its form, and their view of the integration bringing spiritual enjoyment and a peak esthetic experience are regarded as “important but still dissatisfied” by the students. Likewise, this reveals that the application of new media in public art classrooms remains disconnected from the vision, evidenced by its unidimensional form and inability to fully display new media’s characteristics of two-way communication. In the face of this situation, how can new media showcase its advantages and arouse the enthusiasm of all involved?

First, we should clarify that the “new” in new media is being compared with traditional media; the old used to be new, and the new will grow old. The dialectical development between “new” and “old” proves that our current meaning of “new media” will not be an immutable concept. Various “new” things that have evolved and continued throughout the history of media will eventually fall behind and be eliminated in the future. In relation to public art education, universities will need and can have more abundant support from new media in their cause of developing public art education. By continuing to leverage current advantages, new media should also improve new media’s content and form of application by considering the relationship between art, science, and technology. With a more artistic vision and approach, we should contribute to public art education in colleges and universities from the aspects of research and development and application and feedback of new media. This implies that the integration of new media and public art education in colleges and universities will not only bear the nature of art itself but also enable hi-tech media methods to be used to make the educational process more convenient and fun. This will better achieve the goal and realize the significance of public art education in colleges and universities in a bid to cultivate young students’ esthetic and creative ability, and to augment an individual’s soft cultural power for a new generation (Zheng, 2019). Meanwhile, front-line teachers of public art courses should also understand that new media can help educators to innovate teaching ideas, whether online or in-person, or with theoretical or practical teaching. If we wish to give full play to the creativity and open-thinking facet of art itself, and even let artistic thinking play a role like a language expression, the supplement of new media remains indispensable (Martin, 2017). Thus, public art teachers should also keep current with relevant knowledge about new media, hone relevant skills, and make use of new media to achieve better teaching results throughout the teaching process (Paleczek et al., 2022).

#### Direction: From constraining to appealing, from observing to immersing

According to survey results, at present, that consider the problems and challenges encountered in public art education in colleges and universities, on the one hand, new media is oriented in two directions, disciplinary measures and content attraction, which is from the lens of students’ acquisition of knowledge. Many platforms have functions such as roll call, check-in, and homework submission for online or in-person teaching. Nearly half of the students think that this restricts their concentration on the course to a certain degree; while nearly 60% of the students think that screen switching, courseware projection, teacher-student interaction, and other forms of learning have greatly enhanced the appeal factor of the course content to them, helping them master class content (Table 1). On the other hand, from the perspective of artistry, the development of new media is becoming increasingly situation-based and immersive, carrying great significance to art creation and appreciation. Gradually, we get to associate with art through time and space owing to new media technology, placing ourselves in the original atmosphere where art was designed, bringing us as close as possible to the source of art (Sung et al., 2022). Nonetheless, public art education can ride on the favorable conditions in the new media era and back to the place where art was used to cultivate virtues through education.

#### Principles: Put students’ needs first, grounded in esthetic cultivation

The ultimate role of public art education should differ not only from that of various professional courses but also from the art students’ exploration and research of professional art knowledge. Its main purpose is to broaden the horizons of young students through art knowledge and practice, cultivate students’ esthetic taste, and shape their lives and professional study (Zhang, 2021). Therefore, the premise of the integration and innovation of public art education and new media in colleges and universities remains to clearly define the educational purpose and needs of the students. This should all happen under the guidance of educational purposes since teaching content and teaching form should complement each other. The openness of new media imparts a broader artistic vision to the students. Public art education in colleges and universities should start by fostering the esthetic taste of college students, cultivate a positive outlook on life and values, and influence their life and actions by osmosis. By expanding their knowledge and horizons through their ability to think, this will encourage the improvement of professional ability *via* an innovative mind. Accordingly, the evaluation and assessment methods of public art courses can be distinguished from other professional courses. Stemming from the original intention of public art courses and the needs of students, we should provide students with more opportunities for hands-on creation and practice, allowing artistic thinking and esthetic taste to permeate into students’ professional studies and daily life.

### Supported by rich connotations and ready to “firmly stand by”

#### The important thing is to distinguish between the subject and object, while clearly defining media boundaries

To develop public art education in colleges and universities, we must first distinguish between the subject and object, as well as protect artistry itself. Art remains the content of education, so it is the subject. While new media is the means of art education, so it is the object. If, while developing the curriculum, we neglect the content itself because of excessive reliance on new media, the focus of public art education will shift. The ideological and emotional impact of art on students will taper, and the purpose of public art education will not be achieved (Zhang and Yang, 2022). The openness and inclusiveness of new media bring a variety of possibilities to public art classrooms, but also we should stress the appropriate use of new media in public art classrooms, firmly stand by the core of traditional art education, and convey the quintessence of sentiment and thought *via* art. It remains necessary to bolster disciplinary measures in the public art classroom, construct a new media anti-addiction mechanism, and help young students with weaker self-control to properly use new media in public art classrooms to the greatest extent from the perspectives in a rigid or flexible manner.

#### Core: Hold on to traditional elements and boost cultural confidence

What is high-quality teaching content? Young students growing up in the new era will inevitably be misguided when facing a myriad of complex and intermingled art information on the web. This may lead to the blind worship of foreign things rather than remembering the traditional culture of one’s ancestors. Public art education in colleges and universities must be grounded in the new media network era to help young students pay heed to the history of their nation. In the new era, mediocre entertainment plagues us. The amount of knowledge we can gain from traditional gems of antiquity knows no bounds. Content rooted in such remains the cultural deposits that support the development of art education in colleges and universities of various countries and nationalities, as it also provides the foundation for the development of science and technology in the new media era (Kong, 2007; Măduţa, 2014). Only by grounding ourselves in the traditional culture of each nationality, can we boost the self-confidence of national culture, foster a consciousness of national culture, and properly innovate the content and form of art. Only then can art education in colleges and universities advance further at a steady pace in the new media era. Our aim should be to equip the minds of young students with the knowledge and inject vitality into the development of various industries in the country.

#### Bottom line: To reside in safety while having an awareness of danger; guard against cultural invasion

The mission of public art education in the new media era is not only to improve the students’ own ability, but also to have an imperceptible influence on the esthetic appreciation and judgment of the whole nation. New media has opened up the path of cultural exchange with other nations. While having absorbed the quintessence of foreign cultures, inferior, and gaudy culture and arts are also having a hidden influence on the youth of the new era. This implies the interpretation of esthetics is gradually out of our dominance. We are experiencing a “critical battle of civilization” (Qin, 2013). Public art education in colleges and universities should formulate clear goals and focus on fundamental national esthetics and cultural self-confidence. Only by holding firm to this bottom line can the practical applications of public art education in colleges and universities have value. Particularly for the various forms of art born in the new media era, such as film, television, new media art, and more. We also require the right of discourse, not only in art creation but also in art appreciation and art evaluation. This is what we must prioritize, gradually catch up with, and make up for. If basic problems are not dealt with, no matter how rich and diverse the curriculum content is, and no matter how diverse the forms of teaching are, cutting-edge media integration will lose its connotation. Moreover, only when the national art industry develops based on its own cultural accumulation, can the world of art blossom and thrive.

## Conclusion

When public art education in colleges and universities moves toward the new media era, it will not only get propelled by new media, but will also face many challenges (Zhang and Yang, 2022). Public art education in colleges and universities should adhere to its educational purpose. It needs to shoulder the responsibility of cultivating virtues through education in this complicated and dazzling era of new media. It should reap the dividends brought to the cause of art education in the new media era, and follow the developmental pattern of public art education in colleges and universities in this era. By proactively addressing a host of challenges, it will be able to play a leading role in the ideological molding of young students. Based on the analysis of the current integration of public art education and new media in colleges and universities, we have found that the advantages of the integration of new media and art education outweigh its disadvantages. Likewise, it relies on the support and guidance at the policy level, its speed of iteration of new media, and its profound cultural and art resources. The future development of public art education in colleges and universities from the perspective of new media is set to continue to improve. We need to keep pace with the constant change of new media. Similarly, we must perform our duties, continue to innovate, and remain unchanged amid changes. Only then can public art education in colleges and universities begin to improve personal esthetics and advance forward, while protecting national civilization, which will also benefit world art. This is the vested mission of public art education in colleges, universities, and even society (Wang, 2010).

## Author contributions

T-CH and KZ: conceptualization, data curation, writing—original draft preparation, writing—review and editing, and visualization. KZ: methodology, formal analysis, investigation, and funding acquisition. KZ and QT: software. T-CH, KZ, and QT: validation. T-CH and QT: resources, supervision, and project administration. All authors contributed to the article and approved the submitted version.

## Funding

This paper is the accomplishment of “Research on the Implementation and Development Strategy of Public Art Curriculum in Colleges and Universities in the New Era,” a specialized teaching reform and project of “Aesthetic Education” of Southeast University in 2021 (project number: 2021-my-25). This research was also supported by the Fundamental Research Funds for the Central Universities, grant number 3213049408.

## Conflict of interest

The authors declare that the research was conducted in the absence of any commercial or financial relationships that could be construed as a potential conflict of interest.

## Publisher’s note

All claims expressed in this article are solely those of the authors and do not necessarily represent those of their affiliated organizations, or those of the publisher, the editors and the reviewers. Any product that may be evaluated in this article, or claim that may be made by its manufacturer, is not guaranteed or endorsed by the publisher.
